# Supplementary material for: Genome-wide identification, characterization and gene expression of BES1 transcription factor family in grapevine (Vitis vinifera L.)
Source: Sci Rep. 2023 Jan 5;13:240. doi: 10.1038/s41598-022-24407-y (PMC9816167; doi:10.1038/s41598-022-24407-y)
Supplement: Supplementary file 3 — Supplementary Information. [file 41598_2022_24407_MOESM3_ESM.zip › Vvi_Atr/Vitis_vinifera.PN40024.v4.dna_sm.toplevel.fa.vs.Amborella_trichopoda.AMTR1.0.dna_sm.toplevel.fa.html/Atr-AmTr_v1.0_scaffold00171.html]

|  |  |  |  |  |  |  |  |  |  |  |  |  |  |
| --- | --- | --- | --- | --- | --- | --- | --- | --- | --- | --- | --- | --- | --- |
| Duplication depth | Reference chromosome | Collinear blocks | | | | | | | | | | | |
| 0 | Atr-ERN10192 |  |  |  |  |  |  |  |  |  |  |  |  |
| 0 | Atr-ERN10193 |  |  |  |  |  |  |  |  |  |  |  |  |
| 0 | Atr-ERN10194 |  |  |  |  |  |  |  |  |  |  |  |  |
| 0 | Atr-ERN10195 |  |  |  |  |  |  |  |  |  |  |  |  |
| 0 | Atr-ERN10196 |  |  |  |  |  |  |  |  |  |  |  |  |
| 0 | Atr-ERN10197 |  |  |  |  |  |  |  |  |  |  |  |  |
| 0 | Atr-ERN10198 |  |  |  |  |  |  |  |  |  |  |  |  |
| 0 | Atr-ERN10199 |  |  |  |  |  |  |  |  |  |  |  |  |
| 1 | Atr-ERN10200 |  | Vvi-Vitvi16g00002\_t001 |  |  |  |  |  |  |  |  |  |  |
| 1 | Atr-ERN10201 |  | \| \| |  |  |  |  |  |  |  |  |  |  |
| 1 | Atr-ERN10202 |  | \| \| |  |  |  |  |  |  |  |  |  |  |
| 1 | Atr-ERN10203 |  | \| \| |  |  |  |  |  |  |  |  |  |  |
| 1 | Atr-ERN10204 |  | \| \| |  |  |  |  |  |  |  |  |  |  |
| 2 | Atr-ERN10205 |  | \| \| |  | Vvi-Vitvi10g01582\_t001 |  |  |  |  |  |  |  |  |
| 2 | Atr-ERN10206 |  | \| \| |  | \| \| |  |  |  |  |  |  |  |  |
| 2 | Atr-ERN10207 |  | \| \| |  | \| \| |  |  |  |  |  |  |  |  |
| 2 | Atr-ERN10208 |  | Vvi-Vitvi16g00003\_t001 |  | \| \| |  |  |  |  |  |  |  |  |
| 2 | Atr-ERN10209 |  | \| \| |  | Vvi-Vitvi10g01579\_t001 |  |  |  |  |  |  |  |  |
| 2 | Atr-ERN10210 |  | \| \| |  | \| \| |  |  |  |  |  |  |  |  |
| 3 | Atr-ERN10211 |  | \| \| |  | \| \| |  | Vvi-Vitvi16g00015\_t001 |  |  |  |  |  |  |
| 3 | Atr-ERN10212 |  | \| \| |  | \| \| |  | \| \| |  |  |  |  |  |  |
| 3 | Atr-ERN10213 |  | \| \| |  | \| \| |  | Vvi-Vitvi16g04002\_t001 |  |  |  |  |  |  |
| 3 | Atr-ERN10214 |  | \| \| |  | \| \| |  | Vvi-Vitvi16g00013\_t001 |  |  |  |  |  |  |
| 3 | Atr-ERN10215 |  | \| \| |  | \| \| |  | \| \| |  |  |  |  |  |  |
| 3 | Atr-ERN10216 |  | \| \| |  | \| \| |  | \| \| |  |  |  |  |  |  |
| 3 | Atr-ERN10217 |  | \| \| |  | \| \| |  | \| \| |  |  |  |  |  |  |
| 3 | Atr-ERN10218 |  | \| \| |  | \| \| |  | Vvi-Vitvi16g00011\_t001 |  |  |  |  |  |  |
| 3 | Atr-ERN10219 |  | Vvi-Vitvi16g00010\_t001 |  | \| \| |  | Vvi-Vitvi16g00010\_t001 |  |  |  |  |  |  |
| 3 | Atr-ERN10220 |  | \| \| |  | \| \| |  | Vvi-Vitvi16g00005\_t001 |  |  |  |  |  |  |
| 2 | Atr-ERN10221 |  | \| \| |  | Vvi-Vitvi10g01568\_t001 |  |  |  |  |  |  |  |  |
| 2 | Atr-ERN10222 |  | \| \| |  | \| \| |  |  |  |  |  |  |  |  |
| 2 | Atr-ERN10223 |  | \| \| |  | \| \| |  |  |  |  |  |  |  |  |
| 2 | Atr-ERN10224 |  | \| \| |  | \| \| |  |  |  |  |  |  |  |  |
| 2 | Atr-ERN10225 |  | \| \| |  | Vvi-Vitvi10g01563\_t001 |  |  |  |  |  |  |  |  |
| 2 | Atr-ERN10226 |  | \| \| |  | Vvi-Vitvi10g01558\_t001 |  |  |  |  |  |  |  |  |
| 2 | Atr-ERN10227 |  | \| \| |  | Vvi-Vitvi10g01557\_t001 |  |  |  |  |  |  |  |  |
| 2 | Atr-ERN10228 |  | Vvi-Vitvi16g00016\_t001 |  | \| \| |  |  |  |  |  |  |  |  |
| 2 | Atr-ERN10229 |  | \| \| |  | \| \| |  |  |  |  |  |  |  |  |
| 2 | Atr-ERN10230 |  | \| \| |  | Vvi-Vitvi10g02083\_t001 |  |  |  |  |  |  |  |  |
| 2 | Atr-ERN10231 |  | \| \| |  | Vvi-Vitvi10g01555\_t002 |  |  |  |  |  |  |  |  |
| 2 | Atr-ERN10232 |  | Vvi-Vitvi16g00017\_t001 |  | \| \| |  |  |  |  |  |  |  |  |
| 2 | Atr-ERN10233 |  | \| \| |  | \| \| |  |  |  |  |  |  |  |  |
| 2 | Atr-ERN10234 |  | \| \| |  | \| \| |  |  |  |  |  |  |  |  |
| 2 | Atr-ERN10235 |  | Vvi-Vitvi16g00018\_t001 |  | \| \| |  |  |  |  |  |  |  |  |
| 2 | Atr-ERN10236 |  | Vvi-Vitvi16g01489\_t001 |  | \| \| |  |  |  |  |  |  |  |  |
| 2 | Atr-ERN10237 |  | Vvi-Vitvi16g00019\_t001 |  | \| \| |  |  |  |  |  |  |  |  |
| 2 | Atr-ERN10238 |  | \| \| |  | Vvi-Vitvi10g01553\_t001 |  |  |  |  |  |  |  |  |
| 2 | Atr-ERN10239 |  | \| \| |  | \| \| |  |  |  |  |  |  |  |  |
| 2 | Atr-ERN10240 |  | Vvi-Vitvi16g00020\_t001 |  | \| \| |  |  |  |  |  |  |  |  |
| 2 | Atr-ERN10241 |  | Vvi-Vitvi16g00021\_t001 |  | Vvi-Vitvi10g01552\_t003 |  |  |  |  |  |  |  |  |
| 2 | Atr-ERN10242 |  | Vvi-Vitvi16g00022\_t001 |  | \| \| |  |  |  |  |  |  |  |  |
| 1 | Atr-ERN10243 |  |  |  | \| \| |  |  |  |  |  |  |  |  |
| 1 | Atr-ERN10244 |  |  |  | \| \| |  |  |  |  |  |  |  |  |
| 1 | Atr-ERN10245 |  |  |  | Vvi-Vitvi10g01549\_t001 |  |  |  |  |  |  |  |  |
| 1 | Atr-ERN10246 |  |  |  | Vvi-Vitvi10g01548\_t001 |  |  |  |  |  |  |  |  |
